# Free-radical gases on two-dimensional transition-metal disulfides (XS_2_, X = Mo/W): robust half-metallicity for efficient nitrogen oxide sensors

**DOI:** 10.3762/bjnano.9.156

**Published:** 2018-06-05

**Authors:** Chunmei Zhang, Yalong Jiao, Fengxian Ma, Sri Kasi Matta, Steven Bottle, Aijun Du

**Affiliations:** 1 School of Chemistry, Physics and Mechanical Engineering, Queensland University of Technology, Gardens Point Campus, QLD 4001, Brisbane, Australia

**Keywords:** free radical, half-metallicity, nitric oxide (NO), sensors, spin-polarized

## Abstract

The detection of single gas molecules is a highly challenging work because it requires sensors with an ultra-high level of sensitivity. By using density functional theory, here we demonstrate that the adsorption of a paramagnetic unpaired free radical gas (NO) on a monolayer of XS_2_ (X = Mo, W) can trigger the transition from semiconductor to half metal. More precisely, the single-layer XS_2_ (X = Mo, W) with NO adsorbed on it would behave like a metal in one spin channel while acting as a semiconductor in the other spin orientation. The half-metallicity is robust and independent of the NO concentration. In contrast, no half-metallic feature can be observed after the adsorption of other free radical gases such as NO_2_. The unique change in electronic properties after the adsorption of NO on transition-metal sulfides highlights an effective strategy to distinguish NO from other gas species by experimentally measuring spin-resolved transmission. Our results also suggest XS_2_ (X = Mo, W) nanosheets can act as promising nanoscale NO sensors.

## Introduction

Nitrogen oxide (NO*_x_*) gases, one of the most common groups of air pollutants, are known as one culprit of acid rain and can cause serious health issues [1]. For a large-scale monitoring of NO*_x_,* it is highly essential to develop the techniques of toxic gas detection. Up to now, a number of three dimensional (3D) materials, such as nickel phthalocyanine (NiPc) [2], tungsten trioxide (WO_3_) [3], mesoporous silicate [4], composites of nanoflower-like Cu*_x_*O and multilayer graphene (CuMGCs) [5] have been successfully synthesized as new types of room-temperature NO gas sensors.

Compared with 3D materials [6], two-dimensional (2D) materials (sheets with thickness of on atom) [7–15] such as graphene [8,16], phosphorene [9], siligraphene (SiC_5_) [10] and molybdenum disulfide (MoS_2_) [11,17] possess a high specific surface area and high electrical conductivity making them the ideal candidates for gas sensors. In particular, investigations of XS_2_-based (X = Mo, W) monolayer nanodevices demonstrate that they are ultra-sensitive to a number of molecules that are important in environmental studies [18–26]. More specifically, it has been suggested that MoS_2_ exhibits ultrahigh sensitivity to the adsorption of paramagnetic gases such as NO and NO_2_ [11]. Single-layer MoS_2_ has been confirmed to be a good candidate for fabricating field-effect transistor (FET) sensors for NO with high mobility at room temperature [17], and the detection offers a high sensitivity and rapid current response. However, the detailed mechanism regarding the interaction between the MoS_2_ surface and the gas molecules remains unclear. In addition to MoS_2_, 2D WS_2_ is expected to be a more promising material as gas sensor because of its higher thermal stability and wider temperature range of operation [18]. Synthesized FETs layered with WS_2_ are also reported as attractive electronic devices [27]. Besides, the corresponding binding position and energy of NO adsorbed on single-layer MoS_2_ [23] and WS_2_ [28] were analyzed from a theoretical point of view. Even though the gas-sensing properties involving NO and NO_2_ are well studied, few studies [29] have been carried out to explore the difference in spin-polarized gas-sensing between NO and other gas molecules.

A completely spin-polarized current flow can be achieved in a half-metallic material [30–33]. Half-metallic materials exhibit semiconducting behavior in one spin channel and metallic behavior in the opposite spin channel, which could be uses in spintronics. But the lack of suitable materials limits the development of spintronic applications. Doping semiconductors may be applied to achieve spin-polarized currents, but this requires a complicated process. Although many materials have been predicted in theory for this purpose, only few possess all necessary properties and many of them are unstable. Therefore, to explore more experimentally feasible and stable half-metallic materials is highly desirable.

In this paper, a density functional theory (DFT) study is carried out to show that monolayer XS_2_ (X = Mo, W) can selectively detect NO by exhibiting half-metallicity after physical adsorption of gas on the surface. For comparison, an investigation of NO_2_ adsorbed on single-layer XS_2_ (X = Mo, W) is also presented as both NO and NO_2_ are free radicals and can introduce magnetic moments to the gas/XS_2_ (X = Mo, W) system. The binding position and adsorption energy are analyzed in detail. In terms of the projected density of states (PDOS) and orbital contribution, our results offer a deep insight into the Fermi-level pinning mechanism. In addition, we expand the calculations to other 2D layered materials including GaS, GaSe, SnS, SnSe, WSe and Bi_2_Se_3_ (Figure S1, Supporting Information File 1). To the best of our knowledge, our work is the first to propose a NO sensor by detecting spin transmission, which may probe a new prospect for gas sensing.

## Computational Methods

The spin-polarized calculations for NO, NO_2_, and NO, NO_2_ adsorbed on monolayer XS_2_ (X = Mo, W) are performed using DFT within generalized gradient approximation (GGA) of the Perdew–Burke–Ernzerhof (PBE) functional, as implemented in the Vienna ab initio simulation package (VASP) [34–36]. To study 2D systems under the periodic boundary conditions, a vacuum layer with a thickness of more than 20 Å was set to minimize artificial interactions between neighboring layers. A dispersion correction of total energy (DFT-D3 method) [37] is used to incorporate the long-range van der Waals interaction. The structures are fully relaxed until energy and force are converged to 10^−6^ eV and 0.001 eV/Å, respectively. The calculations on band structures and charge density are undertaken with an energy cut-off of 500 eV for the plane-wave expansion and Monkhorst–Pack *k*-point meshes of 3 × 3 × 1 in the whole Brillouin zone. The adsorption energy (binding energy), *E*_ads_ is calculated according to

[1]
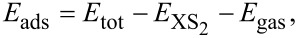


where *E*_tot_ is the total energy with adsorbed NO*_x_* after geometry optimization of the adsorbate; *E*_gas_ is the energy of the adsorbed NO*_x_* molecule after geometry optimization, and 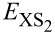 is the energy of 3 × 3 monolayer XS_2_ supercell.

## Results and Discussion

**Figure 1:**
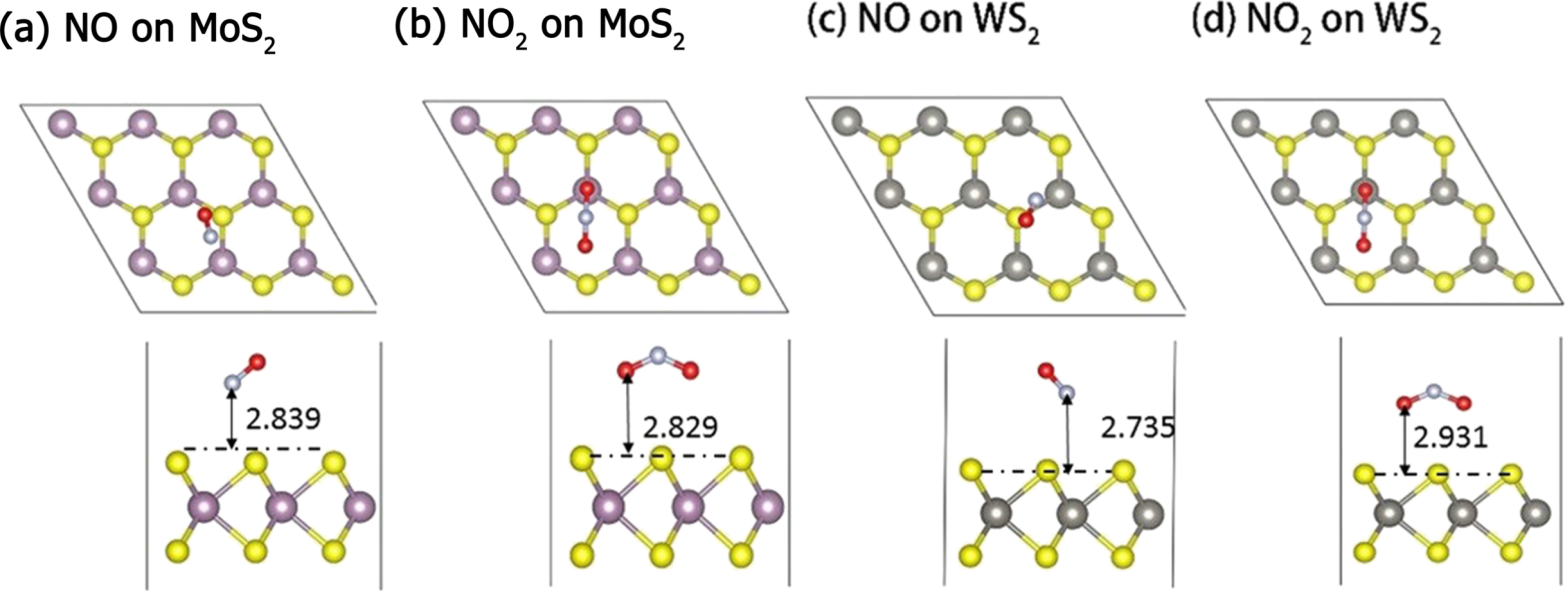
Top and side views of the most favorable NO and NO_2_ adsorption configurations on monolayers of MoS_2_ and WS_2_. The yellow, purple, grey, white and red balls represent S, Mo, W, N, and O atoms, respectively.

We focus our discussions on the adsorption of NO and NO_2_ on XS_2_ (X = Mo, W), as NO and NO_2_ are paramagnetic free radicals. Most other gas molecules, such as NH_3_, CO and CO_2_, have no magnetic moment. Monolayer XS_2_ (X = Mo, W) have a hexagonal configuration, where three pairs of S atoms are anchored on one X atom and form alternating corners (S–X–S) in a honeycomb network. Figure 1 illustrates the top and side view structures of the favorable NO and NO_2_ adsorption position on the 3 × 3 supercell of XS_2_ (X = Mo, W), and Table 1 summarizes the corresponding values of adsorption energy and magnetic moment. The equilibrium height is defined as the vertical distance between N (NO), O (NO_2_) and the top S-layer of the XS_2_ (X = Mo, W) sheet (see Figure 1). The calculation based on a 3 × 3 supercell of monolayer XS_2_, with a single gas molecule adsorbed on it, is chosen as the computational model. A different gas concentration including the model of a unit cell and 6 × 6 supercell with a single gas molecule adsorbed on the surface are also computed (Figure S2, Supporting Information File 1). In Figure 1, the favorable adsorption configuration for NO is the N atom on top of the X–S bond. N is adjacent to S atoms at the monolayer with a distance of 2.839 and 2.735 Å for single-layer MoS_2_ and WS_2_, resulting in adsorption energies of −180 and −165 meV (calculated from Equation 1), respectively. NO_2_ is bonded with the O atoms close to the monolayer with adsorption distances of 2.829 and 2.931 Å, and adsorption energies of −233 and −201meV, respectively, for single-layer MoS_2_ and WS_2_ (Figure 1). An adsorption distance of approximately 3 Å suggests that NO and NO_2_ are physically adsorbed.

**Table 1:** Calculated values of adsorption energy (*E*_ads_) and magnetic moment (*M*) in equilibrium.

	NO		NO_2_	
	*E*_ads_ (meV)	*M* (µ_B_)	*E*_ads_ (meV)	*M* (µ_B_)

MoS_2_	−180	1	−233	1
WS_2_	−165	1	−201	1

**Figure 2:**
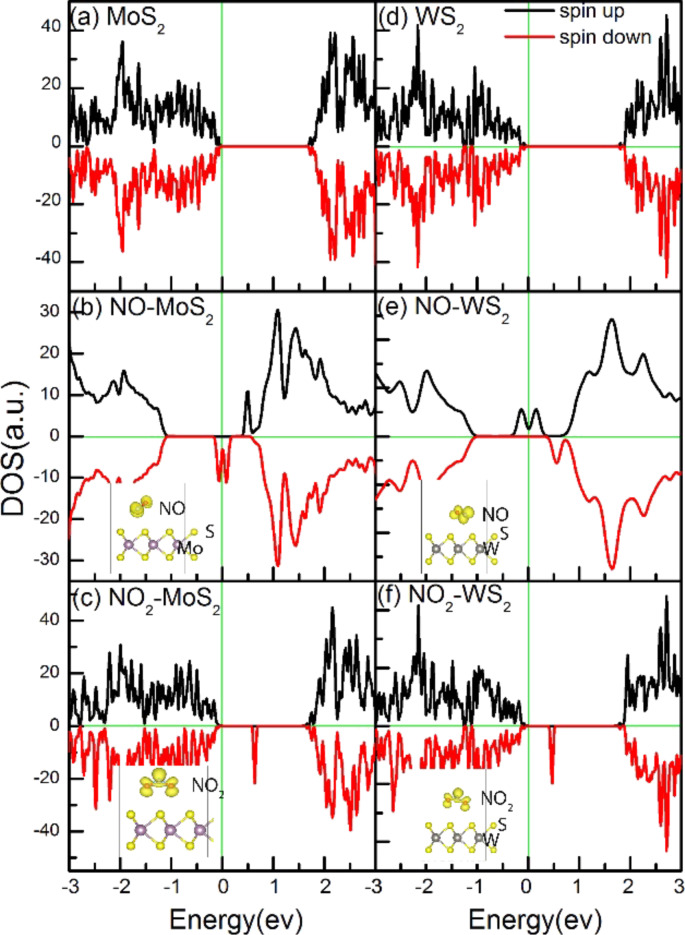
(a–c) TDOS and magnetic charge distribution of (a) the pristine MoS_2_ monolayer, and MOS_2_ with (b) adsorbed NO and (c) adsorbed NO_2_. (d–f) TDOS and magnetic charge distribution of (d) the pristine WS_2_ monolayer, and WS_2_ with (e) adsorbed NO and (f) adsorbed NO_2_. The Fermi level is indicated by the vertical green line.

After determining the most favorable adsorption positions, we calculated the electronic properties after the adsorption of NO and NO_2_. Both NO and NO_2_ are free radical gas molecules with a magnetic moment of 1µ_B_. To fully understand the NO*_x_* adsorption mechanism, it is important to understand the interactions between the monolayer and the adsorbate molecules. Investigations [8,28] revealed that charge transfer occurs from the XS_2_ (X = Mo, W) nanosheet to the physically adsorbed gas molecules. With the calculated favorable configuration, we compute the corresponding magnetic charge density, total density of state (TDOS) as well as PDOS (Figure S3, Supporting Information File 1) for pristine 2D XS_2_ (X = Mo, W) and 2D XS_2_ (X = Mo, W) with adsorbed NO*x* (*x* = 1, 2; Figure 2). Pristine 2D XS_2_ (X = Mo, W) has a band gap of approximately 2 eV. In the case of NO adsorbed on XS_2_ (X = Mo, W), a large density of spin-up electrons appear around the Fermi level, while no spin-down electrons appear. Analyzing the PDOS of a WS_2_ nanosheet with adsorbed NO (Figure S3a–d; Supporting Information File 1), we conclude that the N and O p-orbitals result in absolute spin polarization. In the WS_2_ monolayer with adsorbed NO_2_, no apparent DOS peaks are present around the Fermi level (Figure 2f). The spatial magnetic charge distribution analysis suggests that all the spin-unpaired electrons are localized on the NO and NO_2_ molecule (insets of Figure 2b,c,e,f) [30].

**Figure 3:**
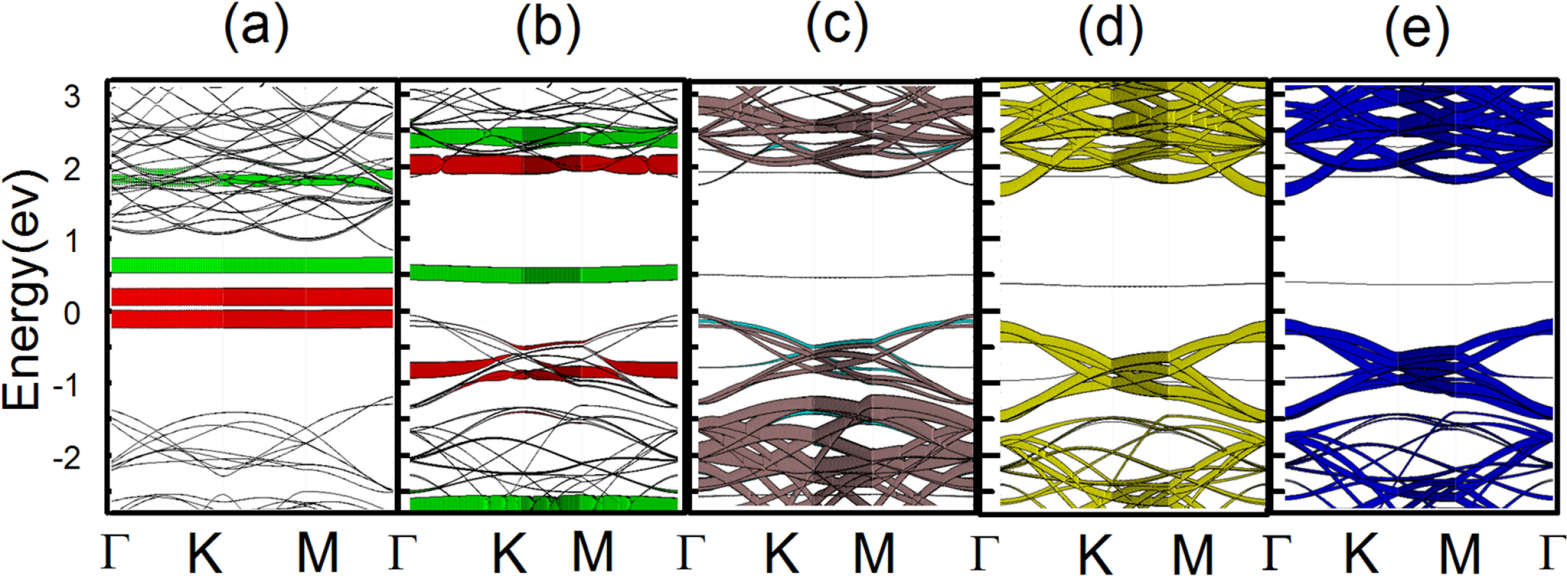
Spin orbital-resolved band structures for WS_2_ with (a) adsorbed NO and (b–e) adsorbed NO_2_. The red and green lines represent the contributions from the spin-up and spin-down p-orbitals of N and O atoms. Cyan lines and brown lines represent the contributions from the spin-up and spin-down p-orbitals of S atoms. Yellow lines and blue lines represent the contributions from the spin-down and spin-up orbitals of W atoms. The Fermi level is set to the energy zero point.

The concept of Fermi-level pinning is usually used in the metal–semiconductor interface region. Zhou et al. [28] Introduced the concept of Fermi-level pinning into the field of gas/2D nanomaterial systems. However, they failed to point out the spin-polarized Fermi-level pinning in gas/2D nanomaterial system. Based on this, we take the single-layer WS_2_ as an example to elucidate the different Fermi-level pinning processes of adsorption of NO and NO_2_. The spin orbital-resolved band structures are simulated (Figure 3) and the Fermi level of the adsorbed gas (NO and NO_2_)/WS_2_ nanomaterial system is pinned around the lowest unoccupied molecular orbital (LUMO) or highest occupied molecular orbital (HOMO) of the adsorbed gas molecules. The PDOS for WS_2_ with adsorbed NO indicates that the W and S atoms have no spin polarization (Figure S3c,d; Supporting Information File 1) and the dominant contribution to the HOMO and the LUMO at the Fermi level originates from the p-orbitals of N and O gas atoms (Figure S3a,b; Supporting Information File 1). Therefore, only the p-orbitals of N and O atoms are plotted in Figure 3. For NO adsorption, only the spin-up p-orbitals (red line in Figure 3a) are pinned around the Fermi level, while the spin-down p-orbitals (green line in Figure 3b) are pinned about 0.5 eV above the Fermi level indicating that the electrical current is completely spin-polarized, which is known as half-metallicity. Therefore, the WS_2_ monolayer with adsorbed NO is ideal for spintronic applications since it has one metallic spin channel and one semiconducting spin channel. After NO_2_ adsorption (Figure 3b), the spin-down and spin-up p-orbitals of the N and O atoms are pinned approximately 0.5 and 2 eV above the Fermi level, respectively. Some impurity states of WS_2_ are pinned slightly below the Fermi level upon adsorption of NO_2_. These impurity states come from the hybridization of spin-up and spin-down orbitals of the W and S atoms (Figure 3c–e), which contribute to the current flow and fail to enable NO_2_/2D WS_2_ system display half-metallicity.

**Figure 4:**
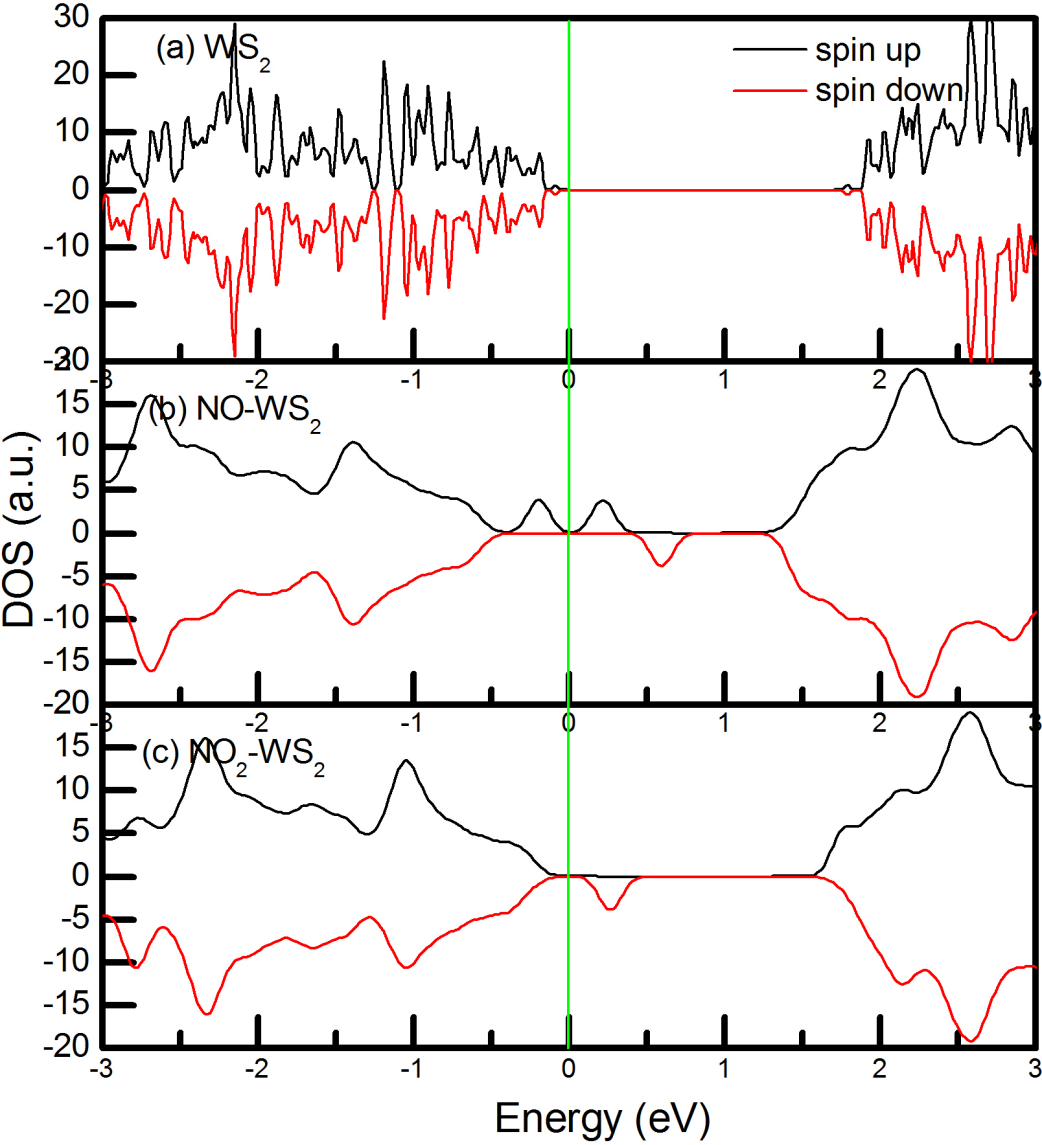
TDOS of (a) the pristine WS_2_ monolayer, and the WS_2_ monolayer with (b) NO adsorbed and (c) NO_2_ adsorbed in humid environment. The Fermi level is indicated by the vertical green line.

In an attempt to address the selectivity towards NO gas in moist environments, we have carried out some new calculations on the adsorption of NO*_x_* (*x* = 1, 2) on the WS_2_ monolayer in the presence of a water molecule. The structures and spin-polarized TDOS for 2D WS_2_ with adsorbed NO*_x_* in humid environment are given in Figure S4 (Supporting Information File 1) and Figure 4, respectively. It can be seen that in humid environment, the spin-polarized current is still presented in the WS_2_ nanosheet with adsorbed NO (Figure 4b) around the Fermi surface, while no apparent spin-polarized current appears when NO_2_ is adsorbed (Figure 4b) or in the pristine WS_2_ monolayer (Figure 4a). This shows that the half-metallic property for WS_2_ with adsorbed NO can be detected even in a humid environment. The water adsorption energy and distance are around −190 meV and 3 Å, suggesting that water is physically adsorbed on the surface of the WS_2_ nanosheet.

Monolayer MoS_2_ [38] and multilayer MoS_2_ field effect transistors [17] for sensing NO at room temperature have been fabricated experimentally. In this work, the ab initio molecular dynamics simulations at room temperature (300 K) for a 3 × 3 supercell model were performed to further evaluate the thermal stability of MoS_2_ with and without the adsorption of NO*_x_* (*x* = 1, 2) as shown in Figure S5 (Supporting Information File 1). There is no significant structural distortion within 7 ps for the adsorption of NO*_x_* onto MoS_2_, indicating highly thermal stability.”

## Conclusion

We have presented monolayer XS_2_ (X = Mo, W) as NO sensor based on first principles studies. The adsorption sites and energy for NO and NO_2_ molecules on MoS_2_ or WS_2_ layers have been studied. The calculations were carried out with different gas concentrations to confirm that the predicted half-metallicity is indeed robust. Differences in electronic properties and orbital analysis between NO and NO_2_ adsorption show that half-metallicity is only observed when NO is adsorbed. Since single/multi-layer MoS_2_ FET sensors for NO have been experimentally obtained, our strategy to detect gas molecules is promising to be applicable.

## Supporting Information

File 1 Additional computational data.
