# Supplementary material for: Free-radical gases on two-dimensional transition-metal disulfides (XS2, X = Mo/W): robust half-metallicity for efficient nitrogen oxide sensors
Source: Beilstein J Nanotechnol. 2018 Jun 5;9:1641–6. doi: 10.3762/bjnano.9.156 (PMC6009531; doi:10.3762/bjnano.9.156)
Supplement: File 1 — Additional computational data. [file Beilstein_J_Nanotechnol-09-1641-s001.pdf]

Supporting Information

for

Free-radical gases on two-dimensional transition-metal disulfides (XS_2, X = Mo/W): robust half-metallicity for efficient nitrogen oxide sensors

Chunmei Zhang, Yalong Jiao, Fengxian Ma, Sri Kasi Matta, Steven Bottle and Aijun Du*

Address: School of Chemistry, Physics and Mechanical Engineering, Queensland University of Technology, Gardens Point Campus, QLD 4001, Brisbane, Australia

Email: Aijun Du* - aijun.du@qut.edu.au

* Corresponding author

Additional computational data

Method

The spin-polarized structural relaxation and electronic property calculations were carried out using density functional theory (DFT) with the Perdew–Burke–Ernzerhof (PBE) generalized gradient approximation [1] exchange–correlation potential as implemented in the Vienna ab initio simulation package (VASP) [2-4]. A dispersion correction of the total energy (DFT-D3 method) [5] was used to incorporate the long-range van der Waals interactions. In addition to MoS_2, and WS_2, we expand our 2D material to GaS, GaSe, SnS, SnSe, WSe and Bi_2Se_3 (Figure S1). A cut-off energy of 500 eV for the plane-wave basis set and a Monkhorst–Pack mesh [6] of 7 × 7 × 1 for the unit cell, 3 × 3 × 1 for the 3 × 3 supercell, and 3 × 3 × 1 for the 6 × 6 supercell for the Brillouin zone integration are employed to find the most favorable adsorption sites for NO and NO_2. For simplicity, only the PDOS of a single NO gas adsorption on unit cell surface is given in Figure S1. The unit cell for a single NO_2 adsorption is not incorporated in order to guarantee a certain distance between two neighbouring NO_2 molecules (Figure S2).

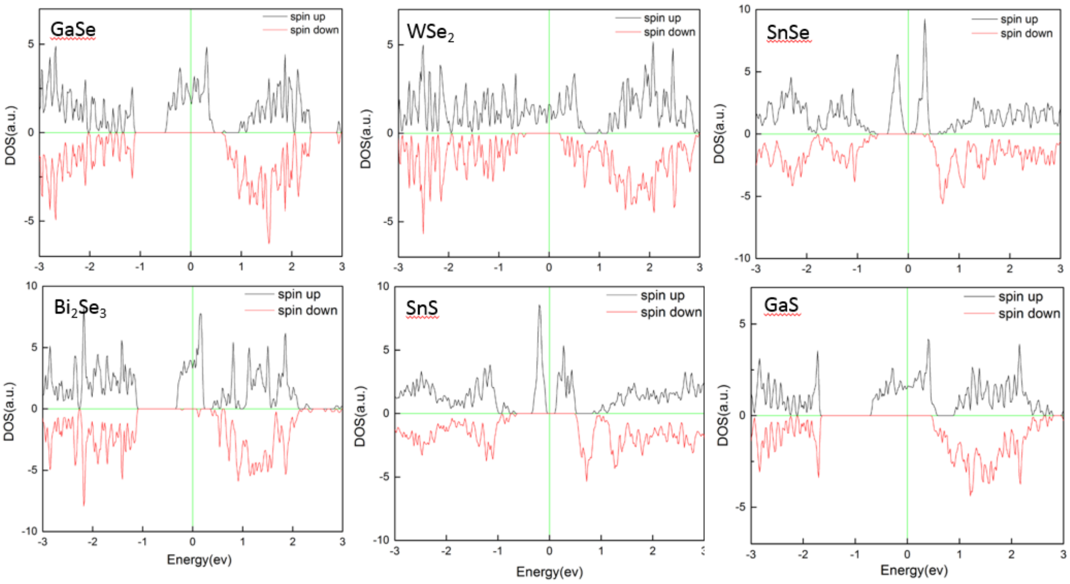

Figure S1: Total density of states (DOS) of NO adsorbed GaSe, WSe_2, SnSe, Bi_2Se_3, SnS and GaS. The Fermi level is indicated by the green line.

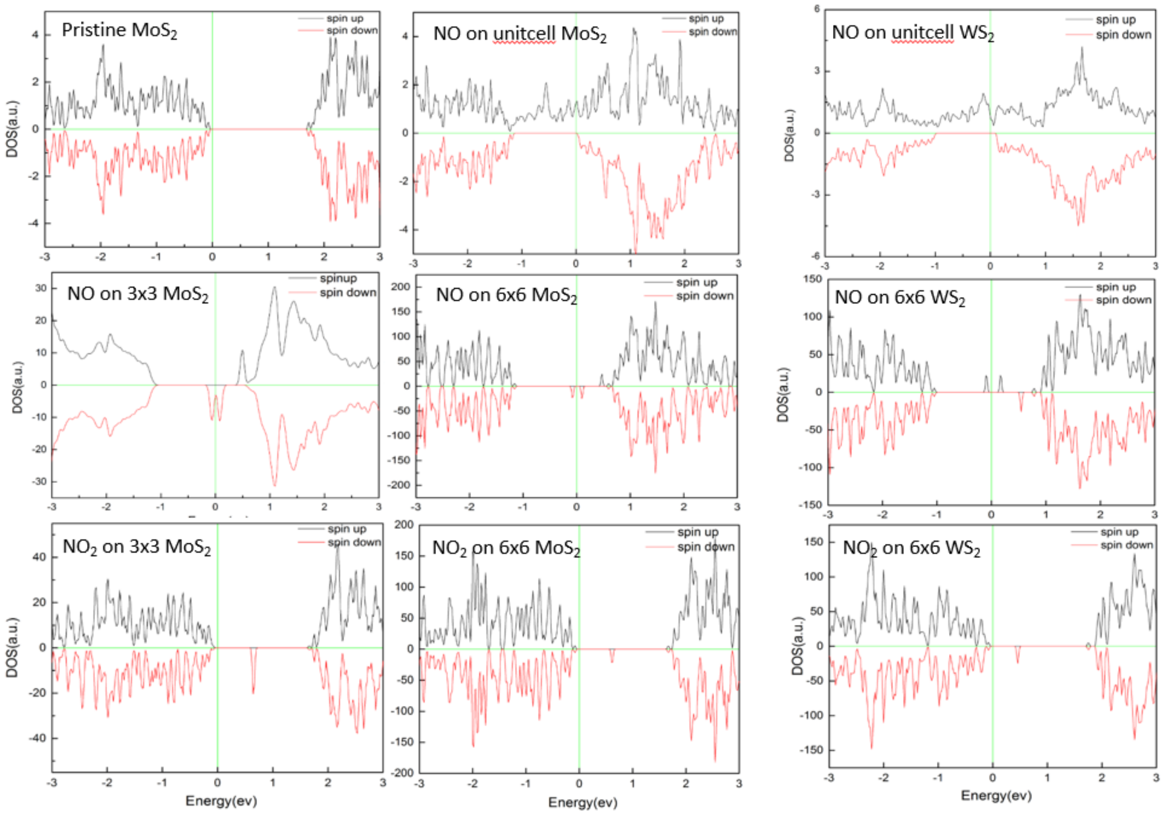

Figure S2: Total density of states (DOS) of the pristine 2D MoS_2 unit cell, 3 × 3 supercell and 6 × 6 MoS_2 supercell and with and with NO*x* (*x* = 1, 2) adsorbed. The Fermi level is indicated by the green line.

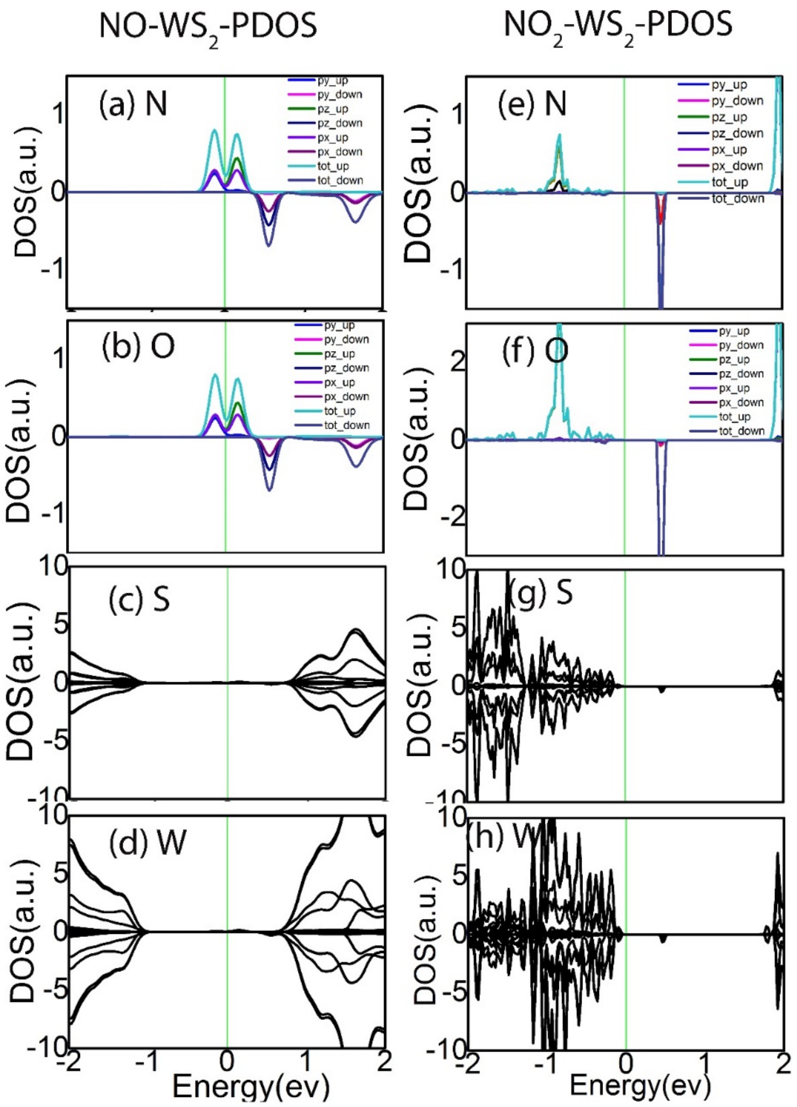

Figure S3: The N, O, W and S PDOS of WS_2 with (a–d) adsorbed NO and with (e–h) adsorbed NO_2. The Fermi level is indicated by the green line.

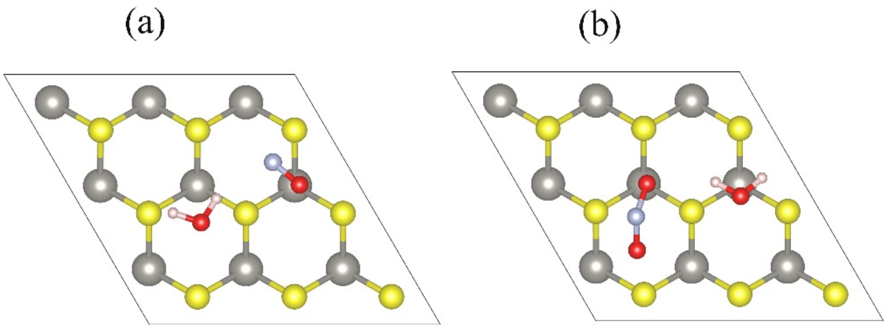

Figure S4: WS_2 with (a) adsorbed NO and with (b) adsorbed NO_2 in humid environment. The white, red, yellow, grey, dark grey balls represent H, O, S, N, W atoms, respectively.

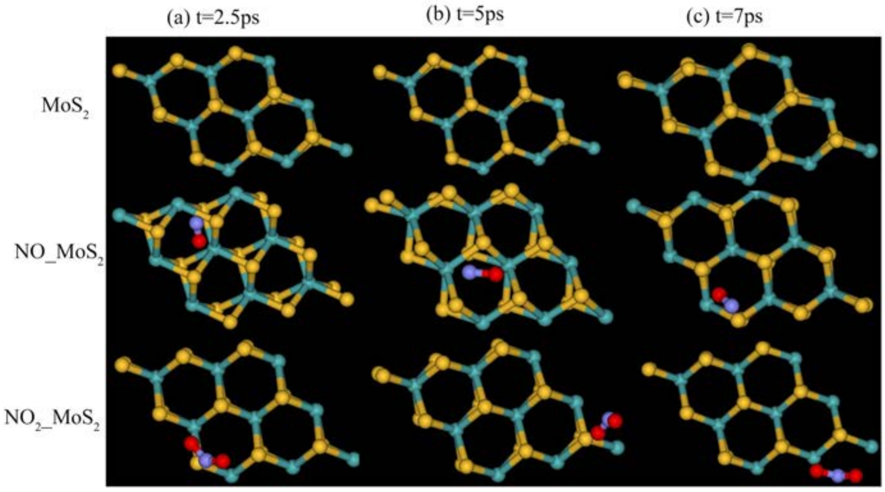

Figure S5: The thermodynamic stability at 300 K of MoS_2 monolayer, NO adsorbed MoS_2 (NO_MoS_2) and NO_2 adsorbed MoS_2 ($NO_2\_MoS_2$) are evaluated through ab initio molecular dynamics (AIMD) simulations. The figures present configurations after (a) 2.5 ps, (b) 5 ps, and (c) 7 ps.

References

1. Perdew, J. P.; Burke, K.; Ernzerhof, M. *Phys. Rev. Lett.* **1996,** *77,* 3865–3868. doi:10.1103/PhysRevLett.77.3865
2. Kresse, G.; Furthmüller, J. *Phys. Rev. B* **1996,** *54,* 11169. doi:10.1103/PhysRevB.54.11169
3. Kresse, G.; Furthmüller, J. *Comput. Mater. Sci.* **1996,** *6,* 15–50. doi:10.1016/0927-0256(96)00008-0
4. Blöchl, P. E. *Phys. Rev. B* **1994,** *50,* 17953. doi:10.1103/PhysRevB.50.17953
5. Grimme, S. *J. Comput. Chem.* **2006,** *27,* 1787–1799. doi:10.1002/jcc.20495
6. Monkhorst, H. J.; Pack, J. D. *Phys. Rev. B* **1976,** *13,* 5188–5192. doi:10.1103/PhysRevB.13.5188
